# From Immunogenic Cell Death to Immunogenic Modulation: Select Chemotherapy Regimens Induce a Spectrum of Immune-Enhancing Activities in the Tumor Microenvironment

**DOI:** 10.3389/fonc.2021.728018

**Published:** 2021-08-23

**Authors:** Kellsye P. Fabian, Benjamin Wolfson, James W. Hodge

**Affiliations:** Laboratory of Tumor Immunology and Biology, Center for Cancer Research, National Cancer Institute, Bethesda, MD, United States

**Keywords:** chemotherapy, immunogenic cell death, immunogenic modulation, cancer, cell stress, immunotherapy

## Abstract

Cancer treatment has rapidly entered the age of immunotherapy, and it is becoming clear that the effective therapy of established tumors necessitates rational multi-combination immunotherapy strategies. But even in the advent of immunotherapy, the clinical role of standard-of-care chemotherapy regimens still remains significant and may be complementary to emerging immunotherapeutic approaches. Depending on dose, schedule, and agent, chemotherapy can induce immunogenic cell death, resulting in the release of tumor antigens to stimulate an immune response, or immunogenic modulation, sensitizing surviving tumor cells to immune cell killing. While these have been previously defined as distinct processes, in this review we examine the published mechanisms supporting both immunogenic cell death and immunogenic modulation and propose they be reclassified as similar effects termed “immunogenic cell stress.”

Treatment-induced immunogenic cell stress is an important result of cytotoxic chemotherapy and future research should consider immunogenic cell stress as a whole rather than just immunogenic cell death or immunogenic modulation. Cancer treatment strategies should be designed specifically to take advantage of these effects in combination immunotherapy, and novel chemotherapy regimens should be designed and investigated to potentially induce all aspects of immunogenic cell stress.

## Introduction

For the past 100 years cancer treatment has undergone rapid evolutions, from surgery to radiotherapy and chemotherapy, to hormonal and molecular targeted therapies and in recent decades immunotherapy. While Paul Ehrlich first hypothesized that the immune system targets nascent tumors for destruction in 1909, it wasn’t until the 1950s that the concept of tumor neo-antigens and the immune surveillance hypothesis was developed (1). The maturation of immunotherapeutic strategies has shed new light on the role of the immune system in effective cancer therapy and reinforced the belief that harnessing the patient’s immune system is a necessary factor in effective cancer treatment. It has also led to the reassessment of the impact of pre-existing cancer treatments, especially radiotherapy and chemotherapy strategies. While different chemotherapeutics have wide-ranging effects on the immune system itself (2), the impact of chemotherapy treatment on tumor immunogenicity and the interplay between tumor and immune response are also significant and may be important for the development of future clinical strategies.

### Origins of Chemotherapy

The 20^th^ century’s evolution of cancer treatment began with the discovery of X rays in 1895, which quickly resulted in their use to treat breast cancer in 1896. The first half of the 20^th^ century saw the rapid discovery of new radiation sources and their application to cancer treatment (3). In combination with surgery, radiotherapy became the standard of care for cancer therapy until the 1960s (4). In parallel with the development of radiotherapy strategies, research that occurred during World Wars I and II resulted in the development of the first chemotherapeutic drugs. Throughout the latter half of the last century novel chemotherapeutic agents were created; to date there are five conventional types of chemotherapy: alkylating agents, antimetabolites, cytotoxic antibiotics, mitotic inhibitors, and topoisomerase targeting agents (5) **(****Table 1****)**.

**Table 1:** Classes of conventional chemotherapy agents.

Class of chemotherapy	Cytotoxic effect	Examples
Alkylating agents I	Abnormal DNA strand crosslinking	Altretamine, aziridine, carboplatin, chlorambucil, cisplatin, cyclophosphamide, dacarbazine, estramustine, ifosfamide, mechlorethamine, melphalan, nitrogen. mustards, oxaliplatin, procarbazine, thiotepa
Antimetabolites	Interfere with metabolic processes, DNA, RNA, protein synthesis	Azacytidine, capecitabine, cladribine, cytarabine, fludarabine, fluorouracil, floxuridine, gemcitabine, hydroxyurea, methotrexate, mercaptopurine, pemetrexed, pentostatin, thioguanine trimetrexate
Cytotoxic antibiotics	Inhibit RNA and DNA synthesis	Actinomycin D, bleomycin, daunomycin, doxorubicin, epirubicin, idarubicin mitoxantrone
Mitotic inhibitors	Disrupt microtubules	Cabazitaxel, docetaxel, paclitaxel, vinblastine, vincristine, vinorelbine
Topoisomerase targeting agents	Prevent DNA replication	Aclarubicin, actinomycin D, camptothecin, daunorubicin, doxorubicin, etoposide, irinotecan, merbarone, mitoxantrone, novobiocin, teniposide, topotecan

Despite their diverse targets and functions, each class of conventional chemotherapy causes cell death through essentially similar mechanisms: they induce DNA damage, disrupt DNA synthesis or repair, or target the basic functions of cell division. However, the diversity of targets means the classes synergize effectively. In the early 1960s the first combination chemotherapy trials were performed, combining nitrogen mustard, vincristine, methotrexate and prednisone (MOMP) or nitrogen mustard, vincristine, prednisone and procarbazine (MOPP). In advanced Hodgkin’s lymphoma, MOPP resulted in complete remission in 80% of patients with no relapse in 60% of patients, and the era of combination chemotherapy was born (4, 6).

### Chemotherapy and the Immune System

By targeting cell division, conventional chemotherapy induces results in the death of rapidly proliferating cells, one of the primary hallmarks of cancerous cells (7). However, by targeting all rapid proliferating cells, chemotherapy also results in patient toxicities and morbidities, including immunosuppression. From the beginnings of chemotherapy research, agents cytotoxic to tumors have also been shown to be immunosuppressive, including nitrogen mustard, which was shown to induce lymphopenia, granulocytopenia and thrombocytopenia early in its development (8). Elimination or depletion of immune cells has remained a frequently observed side effect of many other chemotherapies developed since then. Interestingly, depending on chemotherapy dose and schedule, researchers have shown that chemotherapy-mediated immune cell depletion can be beneficial for the development of a vaccine-stimulated tumor antigen-specific immune response (9–11). Some chemotherapies have also been shown to have direct immunostimulant effects, increasing the maturation and cytotoxic potential of certain immune cell populations (12). In addition to its effects on the immune system itself, chemotherapy has recently been shown to impact the interaction between tumors and the immune system, primarily through cell stress-related processes called immunogenic modulation and immunogenic cell death (ICD).

**Figure 1:**
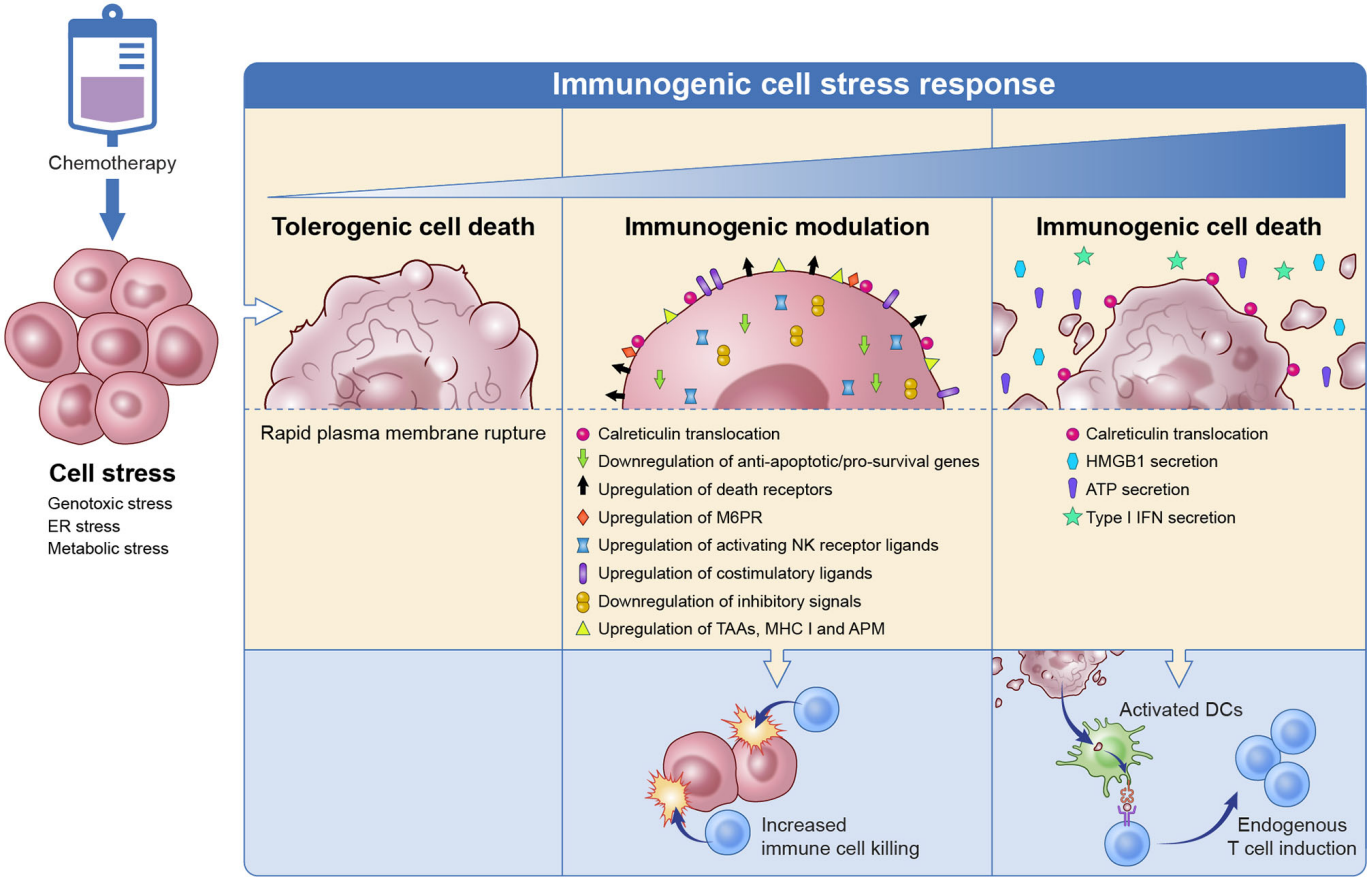
Chemotherapy induces immunogenic cell stress resulting in a spectrum of downstream events from tolerogenic cell death to immune modulation to immunogenic cell death. Tolerogenic cell death induced by chemotherapy is a non-inflammatory cell death that involves the rapid rupture of the plasma membrane and does not trigger immune cells. On the other end of the spectrum, chemotherapy can cause immunogenic cell death that results in the release of DAMPs that promote immune cell phagocytosis, tumor antigen processing, and antigen presentation to T cells. Tumor cells that were not eradicated with chemotherapy undergo immunogenic modulation wherein the tumor surface phenotype is altered to promote T cell targeting of the tumor cell. DAMP, damage-associated molecular patterns; TAA, tumor-associated antigen; MHC, major histocompatibility complex; APM, antigen processing machinery; M6PR, mannose-6-phosphate receptor; NK, natural killer; HMGB1, high mobility group box 1; ATP, adenosine triphosphate; IFN, interferon. Adapted from Ref (14).

The DNA damage, inhibition of DNA synthesis and repair or disruption of cell division caused by chemotherapies damage cells by taking advantage of their cell stress mechanisms (**Figure 1**). If this results in cell death it often induces immunogenic cell death, wherein damage-associated molecular patterns (DAMPs) or “eat me” signals are upregulated to promote immune cell phagocytosis and tumor antigen processing. Common DAMPs include membrane translocation of calreticulin (CRT), release of the pro-inflammatory protein high mobility group box 1 (HMGB1), heat shock protein (HSP) translocation, secretion of adenosine triphosphate (ATP) and type 1 interferons (IFN) (13). Immunogenic cell death and the resulting antigen processing promote a tumor-specific immune system similar to a vaccine. If chemotherapy-induced cell stress does not result in death, it can promote the expression of pro-apoptotic and immune cell engaging molecules such as Fas, tumor necrosis factor (TNF)-related apoptosis-inducing ligand (TRAIL) receptors and increased antigen presentation that sensitize surviving tumor cells to immune cell killing through the process of immunogenic modulation (14). This enables an effective immune response against tumor cells that are resistant to cytotoxic chemotherapy. While immunogenic cell death and immunogenic modulation have previously been believed to be separate processes (14), it is becoming increasingly clear that they are parts of the same spectrum of immunogenic cell stress.

### Effective Combination Immunotherapy

For effective immunotherapy it is necessary to target four different modes of the tumor-immune system interaction. Immunotherapy strategies must induce a tumor antigen-specific T-cell population, expand the number of antigen-specific T cells and promote their migration to the tumor microenvironment, enable a prolonged immune response, and ensure an evolving immune response to prevent tumor escape (15, 16). Depending on the mechanisms engaged, immunogenic cell stress can induce antigen-specific T cells through immunogenic cell death or enable increased immune cell killing of tumor cells through immunogenic modulation. However, as not all chemotherapeutic strategies induce immunogenic cell stress, it is essential for clinicians to rationally design combinations of chemotherapy and immunotherapy for maximum synergy between these two treatment modes. While significant work has investigated these strategies *in vitro* and *in vivo*, few clinical trials currently exist that are explicitly interrogating the immunogenic effects of chemotherapy and how they could support novel immunotherapeutic strategies.

In this review we will discuss the current bodies of work investigating chemotherapy-induced immunogenic modulation and immunogenic cell death and the important similarities and differences in these processes. We posit that, rather than separate processes, immunogenic modulation and immunogenic cell death should be considered as the results of a spectrum of immunogenic cell stress that can be utilized in combination with immunotherapy to develop novel and effective clinical strategies. Finally, we will examine the state of clinical trials investigating the role of immunogenic cell stress and look to the future of combination immunotherapy utilizing standard-of-care agents that both induce immunogenic cell stress and immuno-oncology agents.

## Cell Stress

### The Unfolded Protein Response

Cell stress is the fundamental process governing the induction of chemotherapy-mediated ICD and immunogenic modulation (17, 18). Cell stress is largely governed by the endoplasmic reticulum (ER), an organelle involved in crucial cellular functions such as calcium (Ca^2+^) homeostasis, lipid biosynthesis, and the proper folding and assembly of both secretory and transmembrane proteins (19). Misfolded proteins impede cellular functions, so the quality control performed by the endoplasmic reticulum has evolved to correct misfolded proteins or, if terminally misfolded, destroy them or the cell itself (20). This occurs through the unfolded protein response (UPR), which halts protein translation and increases expression of chaperone proteins to promote proper folding, which if unsuccessful induces cellular apoptosis.

The UPR is facilitated by the ER transmembrane proteins inositol-requiring enzyme 1α (IRE1α), protein kinase R-like ER kinase (PERK), and activating transcription factor 6α (ATF6α) (21). In unstressed cells, these proteins are inactive and are bound to the ER chaperone protein BiP (22–24); however, under conditions of ER stress, BiP dissociates from these UPR sensors to bind misfolded proteins and promote correct folding. These UPR sensors are then free to oligomerize and drive the UPR, halting further protein translation and upregulating stress response gene expression.

While the UPR is an effective quality control process in healthy cells, during tumor development adverse conditions such as accelerated cell division, hypoxia, metabolic stress, and acidosis disrupt the protein folding capacity of the ER (25). This constant sublethal UPR activation allows the tumor cells to resolve misfolded proteins, thereby promoting cell survival and tumor progression in harsh microenvironmental conditions (26). However, the reliance of tumors on the UPR also makes it a tempting target for targeted molecular therapy (27), as well as a potential source for biomarkers of cancer in undiagnosed patients (28, 29).

### Chemotherapy and Cell Stress

While low-level activation of the UPR is essential for tumor survival, intense ER stress, like that caused by chemotherapy, results in the accumulation of the misfolded proteins and ultimately triggers cell death. However, chemotherapeutic agents do not target the ER directly, and do not directly induce the UPR (**Tables 1** and **2**). Immunogenic cell stress inducers such as anthracyclines (doxorubicin and mitoxantrone), oxaliplatin, and cyclophosphamide primarily target the DNA or DNA replication machinery proteins while agents such as cisplatin and taxanes target DNA and microtubules. This frequently results in inhibition of transcription, translation, and cell replication, inducing ER stress through secondary or ‘collateral’ effects (18, 30). In this way chemotherapeutic targeting of an organelle or cell structure of interest also impacts the ER and causes stress *via* mechanisms that have not yet been fully elucidated.

The ER stress stimulated by chemotherapeutic agents results in the activation of all the UPR pathways; however, only the PERK signaling pathway appears to be involved in immunogenic cell stress. Initiation of PERK results in the phosphorylation of the eukaryotic translation initiation factor 2a (eIF2a), which reduces protein translation and influx of newly synthesized proteins into the ER (21, 31, 32). This is followed by the activation of caspase-8, which cleaves the ER-resident membrane protein BAP31, and the activation of pro-apoptotic proteins Bax and Bak (33), which are key players in mitochondrial dysfunction (34). Finally, CRT (a common DAMP as previously mentioned) is translocated from the ER lumen to the plasma surface *via* the Golgi apparatus that is dependent on vesicle-associated membrane protein 1 (VAMP1) and synaptosomal-associated protein 25 (SNAP25) (33). CRT translocation enhances killing of the stressed cell by increasing immune cell recognition, increasing the immunogenicity of the stressed cell (35). Genetic inhibition of *ERN1* (IRE1α) and *ATF6* (ATF6α) did not affect CRT exposure while disruption of the PERK pathway blocked the CRT translocation induced by anthracyclines and oxaliplatin, demonstrating that PERK is mandatory for ICD (33).

The importance of ER stress in chemotherapy-induced immunogenic cell stress has been demonstrated in elegant experiments that blocked ER stress mechanisms and examined the resulting immunogenicity. Treatment with antioxidants inhibits the immunogenicity of dying cells, demonstrating the impact and importance of reactive oxygen-species (ROS)- mediated stress in the immunological activity of some anti-cancer agents (33, 36). Likewise, silencing or pharmacological blocking of molecular components of the ER stress response diminishes the immunogenicity of cell death triggered by ICD-inducing therapies (33, 37), and the intensity and quality of danger signaling during ICD has been shown to be affected by the strength and kinetics of the ER stress induced by anticancer treatments (17, 18).

Immunogenic cell stress is a spectrum of cellular mechanisms ranging from those that sensitize a living cell to immune-mediated killing to those that induce immunogenic cell death. The characteristics of a chemotherapeutic compound that make it capable of inducing immunogenic modulation versus immunogenic cell death remain unknown, and it has been demonstrated that compounds of substantial structural and functional similarity such as oxaliplatin [capable of inducing ICD (38)] and cisplatin [incapable of inducing ICD (39)] have differential immunogenic cell stress effects (40, 41). It is likely that this represents a difference in the levels of cell stress induction; however, further work is necessary to interrogate the continuum of immunogenic cell stress.

## Immunogenic Cell Death

According to the Nomenclature Committee on Cell Death, ICD is “a form of regulated cell death that is sufficient to activate an adaptive immune response in immunocompetent hosts” (42). A variety of cytotoxic chemotherapies have been demonstrated to potentiate the spatiotemporally defined process of ICD, including idarubicin, epirubicin, doxorubicin, mitoxantrone, oxaliplatin, bortezomib, and cyclophosphamide (41).

Chemotherapy-driven ICD is characterized by the secretion or surface exposure of DAMPs by dying tumor cells (13, 43). Cognate receptor binding of DAMPs on antigen-presenting cells (APCs) promotes the recruitment, activation, and maturation of APCs, resulting in the uptake of tumor-associated antigens (TAAs). This is followed by the migration of APCs to the draining lymph nodes where they present the antigens to the cytotoxic T lymphocytes (CTLs), hence stimulating an anticancer response. The best-studied DAMPs pivotal for ICD are CRT, ATP, HMBG1, and type-I IFNs (13, 18, 42).

### Calreticulin

CRT, a Ca^2+^ binding chaperone protein mainly located in the lumen of the ER, is involved in the regulation of Ca^2+^ signaling and homeostasis, as well as major histocompatibility complex (MHC) class I assembly (44). Cells undergoing ICD translocate CRT and its cofactor, ERp57, to the plasma membrane (ecto-CRT) (33). This occurs during the pre-apoptotic stage, prior to caspase-3 cleavage, phosphatidylserine (PS) externalization, and plasma membrane permeabilization (33, 37). Both ecto-CRT and PS provide a potent “eat me” signal to macrophages and dendritic cells (DCs); however, only ecto-CRT triggers ICD while PS mediates the clearance of apoptotic cells and debris without activating an immune response (37, 45–48). Ecto-CRT binds CD91 on the APCs, promoting phagocytosis, tumor antigen presentation and subsequent activation of anti-tumor CTLs. In addition, ligation of ecto-CRT to CD91 on APCs promotes proinflammatory cytokine production and Th17 priming (49). Depletion of CRT using short interfering RNA (siRNA) results in the reduction of oxiplatin-induced immune response, whereas surface adsorption of recombinant CRT restores the immunogenicity of CRT-depleted cells undergoing ICD, implicating the key role of ecto-CRT in determining cell death as immunogenic (37, 38).

### Extracellular ATP

Extracellular ATP is an important molecule involved in numerous autocrine and paracrine cell signaling pathways (50). Similar to CRT translocation, the release of ATP into the extracellular space during chemotherapy-induced ICD typically occurs in the pre-apoptotic stage (51, 52). Pre-mortem autophagy appears to be required (but not sufficient) for the optimal release of ATP as chemotherapy failed to promote ATP secretion and anticancer immune response in autophagy-deficient tumors (52). Chemotherapy activates caspase-dependent secretion of ATP *via* lysosomal exocytosis, plasma membrane blebbing, and pannexin 1 channels (53). Once secreted, extracellular ATP serves as a “find me” signal to APCs *via* ATP binding to the P2Y2 receptor, which directs APC chemotaxis (54, 55). Furthermore, ATP signals through the P2RX7 receptor on the surface of DCs, activates the NLRP3 (NOD-, LRR- and pyrin domain-containing protein 3) inflammasome, and promotes IL-1b production (56). IL-1b stimulates DC activation and antigen presentation, both of which are necessary for the priming of tumor-specific CTLs. In preclinical models, chemotherapy is relatively inefficient against tumors deficient for P2RX7, caspase-1, IL-1b, or IL-1R (56). Furthermore, overexpression of CD39, an ectonucleotidase that hydrolyzes ATP, abrogated the chemotherapy-induced immunogenicity of dying tumor cells (57). In contrast, CD39 blockade improved the anti-tumor activity of immunogenic chemotherapy (58). In the clinical setting, it was observed that breast cancer patients with loss-of-function allele of P2RX7 had unfavorable disease outcomes relative to individuals with the normal allele (56). Altogether, these findings indicate that ATP is a crucial component of ICD-elicited immunogenicity.

### High Mobility Group Box 1 Protein

HMGB1 is a non-histone chromatin-binding protein that is associated with DNA organization and transcription regulation (59). DCs are able to actively secrete their own nuclear HMGB1, where it acts as a signaling molecule for maturation, migration, and polarization of naïve T cells (60, 61). In contrast, HMGB1 is passively released in the extracellular space during the late apoptotic/necrotic stage of ICD, when both the nuclear and plasma membranes have been permeabilized (62). The precise stress response that promotes HMGB1 release is yet to be elucidated (13). Extracellular HMGB1 can bind to different pathogen recognition receptors (PRR) on myeloid cells such as the receptor for advanced glycation end products (RAGE) and Toll-like receptor 4 (TLR4) (63–65). HMGB1-mediated signaling *via* TLR4 signaling through the adaptor protein MYD88 is required and sufficient for ICD based on numerous genetic and pharmacological experiments (64, 66). The HMGB1-TLR4 interaction and downstream signaling inhibit the fusion between phagosome and lysosome, which facilitate processing and cross-presentation of tumor antigens by DCs. Blocking or deletion of critical components of the pathway abrogates chemotherapy-driven ICD (64, 66). Furthermore, breast cancer, colon cancer, and head and neck squamous cell carcinoma patients carrying TLR4 loss-of-function polymorphisms had poorer clinical outcomes with chemotherapy (38, 64, 67).

### Type 1 Interferon

Type I IFNs have classically been strongly linked with antiviral immune responses; recent studies, however, have revealed that they also play a key role in therapy-driven anti-cancer immunity (68). The ICD activity of anthracyclines appears to involve the generation of nucleic acid stress in the form of double-stranded RNA molecules that trigger the endosomal PRR TLR3 (69). TLR3 activation, in turn, stimulates the rapid production of type I IFNs in the malignant cells. Autocrine and paracrine signaling of type I IFN promote the production of CXCL10, which acts as a chemoattractant for T cells. Type I IFN is also important in cancer immunosurveillance and in the cross-priming capacity of APCs (68, 70). Defects in type I IFN signaling impair chemotherapy-driven ICD. Tumors that are genetically deficient for TLR3 or type I IFN receptor are less susceptible to doxorubicin (69). In contrast, expression of *MX1* (a gene signature downstream of type I IFN signaling) is a predictive biomarker for complete response to neoadjuvant anthracycline-based chemotherapy in breast carcinoma. Furthermore, *MX1* expression also predicted metastasis-free survival with neoadjuvant chemotherapy in breast cancer patients with poor prognosis (69).

### Novel ICD-Induced DAMPs

As discussed here and in the previous sections, DAMP emission by cells undergoing ICD is associated with the activation of the intracellular stress response. However, the exact mechanisms of DAMP release and signaling remain to be fully elucidated. Several other DAMPs have also been linked to ICD but have not been extensively described. Anthracycline treatment induces the release of annexin A1 (ANXA1), which mediates the interaction of APCs to the malignant cells undergoing ICD through its cognate receptor formyl peptide receptor 1 (FPR1) (71). Similar to CRT, heat shock proteins (HSP)70 and HSP90 are exposed by chemotherapy and serve as “eat me” signal to DCs (72, 73). Furthermore, mitochondrial transcription factor A (TFAM) is a structural homolog of HMGB1, and during ICD it promotes APC maturation and recruitment *via* the RAGE receptor (74, 75). Future research is likely to identify more immunogenic DAMPs, which may serve as novel biomarkers of immunogenic cell death.

## Immunogenic Modulation

In addition to inducing cell death and the release of DAMPs, chemotherapy can also increase the susceptibility of cancer cells to immune effectors through a process called immunogenic modulation (15, 76). Immunogenic modulation includes alterations in the biology of tumor cells, such as enhanced antigen presentation, changes in surface marker expression, and upregulation of pro-apoptotic molecules (14). This results in sensitizing tumor cells to killing by both CTLs and members of the innate immune response, promoting the destruction of malignant cells that were not eradicated by treatment. This has been demonstrated to result in increased benefit in the clinic. Patients who received chemotherapy and had disease recurrence had higher clinical benefit when treated with immunotherapy compared to individuals who were not previously treated with chemotherapy, demonstrating chemotherapy’s potential to enhance the efficacy of cytotoxic immune effectors (15, 76).

As with immunogenic cell death, immunogenic modulation is not a direct result of chemotherapy, but is instead the result of activation of cell stress pathways. While ICD is the result of chemotherapy-mediated cell stress-inducing cell death, cell stress occurs on a spectrum and does not always result in death of the cell. Immunogenic modulation is therefore the result of an active, but not cytotoxic, cell stress response to chemotherapy. One of the primary functions of cellular immunity is to destroy damaged, infected and malignant cells. Cell stress can prompt both innate and adaptive immune responses through a variety of mechanisms, including upregulation of MHC class I and NKG2D ligands MICA and MICB (77), as well as inducing pro-apoptotic signaling through Fas (78) and TRAIL receptors (79, 80).

### Tumor Antigenicity

Chemotherapy-induced cell stress promotes tumor antigenicity by upregulating the expression and presentation of tumor neoantigens or tumor-associated antigens on the tumor cells. Chemotherapy treatment has been shown to increase the expression of carcinoembryonic antigen (CEA) in colon and breast carcinoma cells (5-fluorouracil and docetaxel) and cancer-testis antigens in renal cell carcinoma and ovarian cancer cells (5′-aza-2′deoxycytidine) (14, 81–83). The observed increase in TAA expression in these studies was also associated with upregulated MHC class I expression and enhanced sensitivity of the chemotherapy-treated neoplastic cells to TAA-specific CTLs. Other chemotherapeutic agents such as cyclophosphamide, oxaliplatin, and gemcitabine have also been found to increase the expression of MHC class I (84). Docetaxel, paclitaxel, and doxorubicin were shown to promote the expression of the components of the MHC class I antigen processing machinery, including calnexin, LMP2, LMP7, TAP1, TAP2 and tapasin in cancer cells (14, 85). Furthermore, chemotherapy treatment can also induce epitope spreading by revealing weaker tumor antigenic epitopes and thus eliciting CTL responses against both dominant and subdominant TAA epitopes (86).

### Sensitization to Immune Attack

Chemotherapy can also alter the surface phenotype of malignant cells to increase tumor susceptibility to CTL attack. Paclitaxel, cisplatin, and doxorubicin treatment rendered tumor cells more sensitive to CTL killing by upregulating mannose-6-phosphate receptors (M6PR) on the tumor cell surface, which augments cell membrane permeability to granzyme B (87). Consequently, antigen-specific CTLs were also able to induce a strong anti-tumor response against neighboring tumor cells that did not express the tumor antigens. Chemotherapy also has the capability of inducing the expression of costimulatory molecules such as CD80 and inhibiting the expression of checkpoint molecules PD-L1 and PD-L2 on the tumor cell surface, resulting in enhanced recognition and killing by CTLs (88–90).

Innate immune cell activity against tumor cells can also be promoted with chemotherapy. In addition to providing danger signal during ICD, ecto-CRT has also been associated with improved IL-15 trans-presentation to natural killer (NK) cells (91). Hence, chemotherapy-induced CRT translocation to the plasma membrane may also sensitize the tumor cells to NK cell cytolysis. Chemotherapeutic agents have also been shown to sensitize tumor cells to NK cell cytolysis through the induction of ligands on the tumor surface such as MICA/B, ULPBs and B7-H6 that bind activating NK receptors (92).

### Disruption of Survival Signaling

In addition to promoting increased interaction between tumor cells and cytotoxic immune cells, chemotherapy can also upset the pro-survival signaling in the tumor cells. Different chemotherapies have been shown to stimulate the expression of death receptors on the surface of tumor cells (93), including FAS (also known as CD95), and TRAIL-R1 and TRAIL-R2 (also known as DR4 and DR5) on a large panel of cancer cells. When these death receptors interact with their cognate ligands, apoptosis is triggered. Hence the upregulation of death receptors may render cancer cells more susceptible targets for NK and T cells that express and secrete death ligands, thereby resulting in improved immune clearance (93). Conversely, anti-apoptotic and/or pro-survival genes, such as those belonging to the Bcl-2 gene family, have been shown to be downregulated by chemotherapy (94, 95).

Taken together, these observations indicate an immunogenic role for chemotherapy that, while distinct from ICD, is a result of cellular stress pathways. This is particularly advantageous since not all cancer cells can be eradicated with chemotherapy. Immunogenic modulation to sensitize malignant cells to adaptive and innate immune attack is an additional layer of chemotherapy-mediated immunogenic cell stress that can be exploited in the clinic for novel chemotherapy-immunotherapy combination therapy.

## Immunogenic Cell Stress in the Clinic

While chemotherapy-mediated immunogenic cell stress has been demonstrated consistently in the laboratory, few studies have investigated its effects in the clinic. Clinical progress has been hampered by the ongoing studies for the most effective chemotherapy dose and schedule to induce immunogenic cell stress, lack of systemic biomarkers and difficulty obtaining appropriate patient samples (96). However, as immunotherapy strategies are maturing, more trials are investigating the direct immunogenic effects of standard-of-care chemotherapy as well as novel chemotherapy treatment strategies to determine the best methods for these proven agents to effectively synergize with immuno-oncology agents. For example, preliminary data from the CheckRad-8 study (NCT03426657) demonstrated that induction treatment with a single cycle of cisplatin and docetaxel combined with durvalumab and tremelimumab resulted in pathologic complete response in the rebiopsy (48%) and increased intratumoral CD8+ cells (45%), indicating the feasibility and antitumor activity of this chemo-immunotherapy combination (97). Two recent review papers have identified 161 ongoing or recently completed clinical trials utilizing at least one chemotherapeutic agent that has been previously demonstrated to induce ICD (41, 98). Furthermore, we recently published an additional review discussing clinical trials that include immunogenic modulation and immunogenic cell death inducing agents in combination immunotherapy strategies on a cancer vaccine backbone (15).

### Chemotherapy Dose and Schedule

One of the most important considerations when utilizing immunogenic cell stress in clinical strategies is the optimal chemotherapy dose and schedule to induce immunogenic cell stress and to combine it with immuno-oncology agents. Most chemotherapeutic agents are used at or near their maximum tolerated dose, which although effective at inducing tumor cell death often results in significant host toxicity, including immune suppression (99). To combat this, alternative chemotherapy dosing schedules have been investigated. The most common alternative is metronomic chemotherapy, a strategy wherein frequent low doses of chemotherapy are delivered to the patient. Metronomic chemotherapy is better tolerated, especially by older or infirm patients, and while it still has been shown to deplete immune cells in certain contexts (100), it also induces immunogenic cell death (85, 101). Some have also proposed the use of medium-dose intermittent chemotherapy, wherein chemotherapy is given at a dose high enough to be cytotoxic in a majority of tumor cells, but not so high that it induces significant immunosuppression (102, 103). These different doses and schedules have been reviewed in detail previously (102, 104, 105), and therefore will not be here. Medium-dose intermittent chemotherapies have yet to enter the clinic, and while there are approximately 130 clinical trials examining metronomic chemotherapy (clinicaltrials.gov), we have identified nine trials utilizing metronomic chemotherapy in immunotherapy combination strategies (**Table 2**). To date no data have been published.

**Table 2:** Clinical trials combining metronomic chemotherapy with immuno-oncology agents.

NCT number	Trial title	Conditions	Treatment	Phases
NCT03971045	Pembrolizumab and Oral Metronomic Cyclophosphamide in Patients With Chest Wall Breast Cancer	Breast cancer Chest wall tumor	Pembrolizumab	Phase 2
NCT03801304	Trial to Evaluate Safety and Efficacy of Vinorelbine With Metronomic Administration in Combination With Atezolizumab as Second-line Treatment for Patients With Stage IV Non-small Cell Lung Cancer	Non-small cell lung cancer	Atezolizumab, vinorelbine	Phase 2
NCT03387111	QUILT-3.090: NANT Squamous Cell Carcinoma (SCC) Vaccine: Subjects with SCC Who Have Progressed	Squamous cell carcinoma	Aldoxorubicin HCl, ETBX-011, ETBX-021, ETBX-051, ETBX-061,GI-4000, GI-6207, GI-6301, haNK for infusion, avelumab, bevacizumab, capecitabine, cetuximab, cisplatin, cyclophosphamide, fluorouracil, leucovorin, nab-paclitaxel, necitumumab, SBRT, N-803	Phase 1/ Phase 2
NCT03387098	QUILT-3.070: Pancreatic Cancer Vaccine: Subjects With Pancreatic Cancer Who Have Progressed on or After Standard-of-care Therapy	Pancreatic cancer	Aldoxorubicin HCl, ALT-803, ETBX-011, GI-4000, haNK for infusion, avelumab, bevacizumab, capecitabine, cyclophosphamide, fluorouracil, leucovorin, nab-paclitaxel, lovaza, oxaliplatin, SBRT	Phase 1/ Phase 2
NCT03329248	QUILT-3.060: NANT Pancreatic Cancer Vaccine: Molecularly Informed Integrated Immunotherapy in Subjects With Pancreatic Cancer Who Have Progressed on or After Standard-of-care Therapy	Pancreatic cancer	ALT-803, ETBX-011, GI-4000, haNK for infusion, avelumab, bevacizumab, capecitabine, cyclophosphamide, fluorouracil, leucovorin, nab-paclitaxel, lovaza, oxaliplatin, SBRT	Phase 1/ Phase 2
NCT03136406	QUILT-3.039: NANT Pancreatic Cancer Vaccine: Combination Immunotherapy in Subjects With Pancreatic Cancer Who Have Progressed on or After Standard-of-care Therapy	Pancreatic cancer	Cyclophosphamide, oxaliplatin, capecitabine, 5-fluorouracil|, leucovorin, nab-paclitaxel, bevacizumab, avelumab, ALT-803, aNK for infusion, ETBX-011, GI-4000	Phase 1/ Phase 2
NCT02998983	Racotumomab in Patients With High-risk Neuroblastoma	Neuroblastoma	Racotumomab	Phase 2
NCT01192555	Allogeneic Tumor Cell Vaccination With Oral Metronomic Cytoxan in Patients With High-Risk Neuroblastoma	Neuroblastoma	Neuroblastoma vaccine, cytoxan	Phase 1/ Phase 2
NCT01159288	Trial of a Vaccination With Tumor Antigen-loaded Dendritic Cell-derived Exosomes	Non-small cell lung cancer	Dex2	Phase 2

NCT number: clinicaltrials.gov identification number. ETBX-011: adenoviral CEA vaccine, ETBX-021: adenoviral HER2 vaccine, ETBX-051: adenoviral brachyury vaccine, ETBX-061: adenoviral MUC1 vaccine, GI-4000: yeast Ras vaccine, GI-6207: yeast CEA vaccine, GI-6301: yeast brachyury vaccine, haNK: high avidity NK cell, SBRT: stereotactic body radiation therapy, N-803 (ALT-803): superagonist interleukin-15:interleukin-15 receptor alphaSu/Fc fusion complex, Dex2: tumor antigen loaded dendritic cell-derived exosomes.

### Clinical Biomarkers

Several studies have been published examining defined immunogenic cell stress biomarkers in the clinic. In lung cancer patients, investigators found that serum levels of CRT, an aforementioned DAMP indicative of cell stress, were significantly increased compared to healthy controls, and further overexpressed in lung cancer patients who had received chemotherapy compared to those who had not (chemotherapy type not specified) (106). However, no comparison between serum calreticulin and patient response was made. In ovarian carcinoma and non-small cell lung cancer (NSCLC), chemotherapy-independent CRT exposure was associated with increased immune cell infiltrates in the tumor and superior overall survival (107, 108), potentially lending clinical significance to the importance of CRT translocation in immunogenic cell stress. In acute myeloid leukemia (AML), researchers found that translocation of calreticulin and upregulation of HSP70 and HSP90 were not increased by treatment with the immunogenic cell death-inducing agents anthracycline, idarubicin or daunorubicin; however, they did find an increase in calreticulin exposure in AML blasts compared to healthy controls. Moreover, patients with high calreticulin exposure had improved disease outcome, regardless of chemotherapy, compared to calreticulin low patients. Interestingly, the investigators also reported a decrease in serum levels of the immunogenic cell stress marker HMGB1 following chemotherapy treatment, which they hypothesized might have been due to decreased numbers of abnormal cells releasing HMGB1 (109). Serum HMGB1 has also been shown to decrease following treatment in breast cancer, and was demonstrated to correlate with treatment efficacy (p=0.053) (110). However, a separate study reported that complete loss of HMGB1 is linked to poor response in breast cancer. Immunohistochemistry examination of breast cancer tumors from patients treated with adjuvant anthracycline showed that tumors with no nuclear HMGB1 staining in the majority of their cells were significantly associated with a negative impact on overall and progression-free survival (111).

These findings make it clear that further investigation into effective biomarkers and the dynamics of immunogenic cell stress in the clinic is required to validate published pre-clinical findings. We have identified five clinical trials (**Table 3**) focused on observation and biomarker detection. The first, a completed study of patients with non-small cell lung cancer (NCT02921854) is looking for exosomal or molecular markers of ICD in the serum of patients following high-dose radiotherapy or concurrent cisplatin and radiotherapy. However, no data are available at this time. An additional completed trial (NCT01513408) is investigating biomarkers in non-small cell lung cancer; no results have been reported yet.

**Table 3:** Clinical trials investigating immunogenic cell death.

NCT number	Trial title	Conditions	Treatment	Phases
NCT01513408	Relevance of T Lymphocytes Tumor Infiltrates CD8 and Foxp3 as Immune Prognostic Biomarker in Breast Cancer Treated by Neo Adjuvant Chemotherapy	Breast cancer	n/a	n/a
NCT02921854	Detection of Circulating Biomarkers of Immunogenic Cell Death	Non-small cell lung cancer	n/a	n/a
NCT01516710	Oslo Randomized Laparoscopic Versus Open Liver Resection for Colorectal Metastases Study	Secondary malignant neoplasm of liver Colorectal neoplasms	n/a	n/a
NCT04256616	Immunogenic Cell Death as a Novel Mechanism of Mitomycin C Activity in Bladder Cancer	Bladder cancer	n/a	n/a
NCT03942900	Immunomonitoring and Biomarker Research in Patients With Squamous Cell Anal Carcinoma	Anal canal cancer	n/a	n/a
NCT01666444	VTX-2337 and Pegylated Liposomal Doxorubicin (PLD) in Patients With Recurrent or Persistent Epithelial Ovarian, Fallopian Tube or Primary Peritoneal Cancer	Epithelial ovarian cancer; Fallopian tube cancer; Primary peritoneal cancer	VTX-2337, pegylated liposomal doxorubicin	Phase 2
NCT01637532	Feasibility of the Combination of Chemotherapy (Carbo/Caelyx or Carbo/Doxorubicin) With Tocilizumab (mAb IL-6R) and Peg-Intron in Patients With Recurrent Ovarian Cancer	Recurrent ovarian cancer	Carboplatin and caelyx or doxorubicin, tocilizumab and interferon alpha 2-b	Phase 1/ Phase 2
NCT03186326	Standard Chemotherapy vs Immunotherapy in 2nd Line Treatment of MSI Colorectal Metastatic Cancer	Metastatic colorectal cancer Microsatellite instable (MSI)	FOLFOX, FOLFIRI, avelumab, panitumumab, cetuximab, bevacizumab, aflibercept	Phase 2
NCT03276013	Pembrolizumab in Combination With Doxorubicin in Advanced, Recurrent or Metastatic Endometrial Cancer	Endometrial Neoplasms	Doxorubicin, pembrolizumab	Phase 2
NCT03409198	Phase IIb Study Evaluating Immunogenic Chemotherapy Combined With Ipilimumab and Nivolumab in Breast Cancer	Breast cancer Hormone receptor positive tumor Metastatic breast cancer	Ipilimumab, nivolumab, pegylated liposomal doxorubicin, cyclophosphamide	Phase 2
NCT03721653	FOLFOXIRI + Bev + Atezo vs FOLFOXIRI + Bev as First-line Treatment of Unresectable Metastatic Colorectal Cancer Patients	Metastatic colorectal cancer	FOLFOXIRI, bevacizumab, atezolizumab	Phase 2
NCT03164993	Atezolizumab Combined With Immunogenic Chemotherapy in Patients With Metastatic Triple-negative Breast Cancer	Breast cancer Triple-negative breast cancer	Atezolizumab, pegylated liposomal doxorubicin, cyclophosphamide	Phase 2
NCT03388190	METIMMOX: Colorectal Cancer METastasis - Shaping Anti-tumor IMMunity by OXaliplatin	Metastatic colorectal cancer	FLOX, nivolumab	Phase 2
NCT03321643	Atezolizumab, Gemcitabine, Oxaliplatin, and Rituximab in Treating Patients With Relapsed or Refractory Transformed Diffuse Large B-Cell Lymphoma	Recurrent diffuse large B-cell lymphoma; Recurrent transformed non-Hodgkin’s lymphoma; Refractory diffuse large B-cell lymphoma; Refractory transformed non-Hodgkin’s lymphoma; Richter syndrome; Transformed follicular lymphoma to diffuse large B-cell lymphoma	Atezolizumab, gemcitabine, oxaliplatin, rituximab	Phase 1
NCT04043195	Nivolumab and Ipilimumab in Combination With Immunogenic Chemotherapy for Patients With Advanced NSCLC	Advanced non-small cell lung cancer (NSCLC)	Oxaliplatin, nivolumab, ipilimumab	Phase 1/ Phase 2
NCT04463368	Isolated Hepatic Perfusion in Combination With Ipilimumab and Nivolumab in Patients With Uveal Melanoma Metastases	Uveal melanoma Liver metastases	Melphalan, ipilimumab, nivolumab	Phase 1
NCT04072263	Adoptive T Cell Therapy in Patients With Recurrent Ovarian Cancer	Recurrent ovarian cancer	Tumor-infiltrating lymphocytes, interferon alfa2A, carboplatin, paclitaxel	Phase 1/ Phase 2
NCT04262687	Chemotherapy and Immunotherapy as Treatment for MSS Metastatic Colorectal Cancer With High Immune Infiltrate (POCHI)	Metastatic colorectal cancer; High immune infiltrate; Microsatellite stable (MSS)	Capecitabine, oxaliplatin, bevacizumab, pembrolizumab	Phase 2

VTX-2337: TLR8 agonist, FOLFOX: chemotherapy regimen of folinic acid, fluorouracil and oxaliplatin, FOLFIRI: chemotherapy regimen of folinic acid, fluorouracil and irinotecan, FOLFOXIRI: chemotherapy regimen of folinic acid, fluorouracil, oxaliplatin and irinotecan, FLOX: chemotherapy regimen of folinic acid, fluorouracil and oxaliplatin.

One active observational trial investigated patients with liver metastases from colorectal carcinoma (NCT01516710). While the main purpose of the study was to compare laparoscopic versus liver resection techniques for identifying colorectal metastases, secondary outcomes included investigating the metastases for markers of ICD. Using deep sequencing, the authors found that patients who had received neoadjuvant chemotherapy (which included known ICD inducer oxaliplatin in 11/15 patients) had a gene signature including genes related to toll-like receptor signaling, IFN response and leukocyte infiltration (112). In a further study investigating this patient population, it was shown that while there was no association between neoadjuvant chemotherapy and intratumoral T-cell density within colorectal liver metastases, there was a significant increase in intratumoral T-cell density in patients who received neoadjuvant chemotherapy fewer than 9.5 weeks before liver metastases resection compared to both patients with a longer interval and those who did not receive chemotherapy. This result is highly interesting and could be an important data point when designing clinical strategies meant to take advantage of chemotherapy-induced immunogenic cell death (113).

### Immunogenic Cell Stress in Therapeutic Strategies

Although it is undeniable that chemotherapeutic agents induce ICD and immunogenic modulation, it remains unclear how these processes contribute to the clinical efficacy of chemotherapies. Clues to the importance of immunogenic cell stress in attaining chemotherapy-mediated therapeutic benefit can be gleaned from a randomized phase III trial comparing upfront oxaliplatin and 5-fluorouracil combination to sequential chemotherapy with single agent 5-fluorouracil until failure, followed by oxaliplatin and 5-fluorouracil combination in colorectal cancer (NCT00126256). In this study, oxaliplatin treatment increased the progression-free survival and overall survival of patients with normal TLR4 allele but did not improve clinical outcomes in patients with loss-of-function TLR4 allele. Without chemotherapy treatment, no differences in disease-free survival were observed among patients with normal or variant TLR4 allele. The results show that TLR4, a receptor for HMGB1, may be a prognostic factor but only in the context of immunogenic chemotherapy. The data indicate that immunogenic cell stress, specifically ICD, may indeed be a contributor to the clinical efficacy of oxaliplatin (38).

We identified an additional 13 clinical trials (**Table 3**) explicitly examining chemotherapy-induced immunogenic cell death, including two that are completed, four that are active and seven that are currently recruiting or yet to begin. Only one of the completed trials has published the complete results of the trial. This phase II trial (NCT01666444) investigated the combination of the toll-like receptor 8 (TLR8) agonist motolimod with ICD-inducing chemotherapeutic pegylated liposomal doxorubicin in women with recurrent or persistent ovarian cancer. Motolimod has previously been shown to activate NK cells, promote antibody-dependent cellular cytotoxicity, increase IFNγ production and drive the maturation of dendritic cells. Therefore, investigators hypothesized that motolimod treatment would synergize with the induction of ICD in patients. The investigators found that while motolimod combined with doxorubicin was well tolerated, there was no significant improvement in overall survival or progression-free survival compared to placebo. They observed that despite the lack of efficacy, motolimod did increase plasma expression of inflammatory cytokines and chemokines, including IL-1β, IL-6 and TNF-⍺. Interestingly, patients who experienced injection site reaction were determined to have a longer overall survival (19.8 months) than those who did not (13.3 months). It is possible this is demonstrative of overall patient immune response, and may indicate the need for patient selection for treatment with motolimod in this context (114).

A second phase I/II trial in ovarian cancer investigated the combination of the ICD inducing chemotherapeutics carboplatin or doxorubicin in combination with tocilizumab, an anti-IL-6R antibody, and pegylated IFN-⍺ (NCT01637532). IL-6 promotes the polarization of macrophages into immunosuppressive M-2 like macrophages and has been demonstrated to recruit T-regulator (Treg) cells, both of which work to inhibit an anti-tumor immune environment. Furthermore, IFN-⍺ promotes DC maturation. The investigators hypothesized that in combination with ICD, anti-IL-6 and IFN-⍺ would promote an anti-tumor immune reaction. While the complete data have yet to be published, this trial reported no dose-limiting toxicities (DLTs), and found that IL-6 was effectively blocked when 8mg/kg tocilizumab was delivered (115). The survival results from the phase II portion of the trial have yet to be released.

The efficacy of inducing immunogenic cell stress and immunogenic cell death in the clinic remains to be verified but immunogenic cell stress inducing agents will remain key parts of cancer treatment. It is likely they will play a role in the development of rationally designed immunotherapy strategies in the future.

## Conclusion

In many indications chemotherapeutic agents remain the standard-of-care therapy despite their high potential for toxicity. While the discovery of immuno-oncology agents has resulted in a revolution in cancer treatment, clinicians have also realized that monotherapy treatment is insufficient in many patients. This has led to the development of combination immunotherapy treatment strategies and, importantly, to the rational combination of immuno-oncology agents with standard-of-care chemotherapy agents. While these often synergize well, with further research into the mechanisms and optimum dose/schedule by which chemotherapy agents induce immunogenic cell stress, they may be applied in highly effective targeted combinations with existing and future immuno-oncology agents.

Significant work has demonstrated that many chemotherapy agents can promote either immunogenic modulation or immunogenic cell death. It has become increasingly clear that these are the result of the same mechanistic pathways underneath the umbrella of immunogenic cell stress. Through cell stress mechanisms, chemotherapy sensitizes tumor cells to immune cell killing and increases the likelihood of tumor antigens released by dead tumor cells stimulating the immune system, resulting in increased numbers and infiltration of tumor-specific T cells and other immune cells necessary for immune-mediated tumor resolution.

While the cell stress response is a necessary mechanism for cellular health and quality control in response to intrinsic errors, there is a wide spectrum of cell stress responses depending on the level and duration of the stress. In the case of cells treated with cytotoxic chemotherapy, this means that the specifics of the cell stress response are dependent on the utilized dose, number of treatments, and other factors (116). However, the full dynamics of the cell stress response in relation to chemotherapy remain unknown, and further investigation is necessary to better enable clinicians to strategically utilize chemotherapy to induce immunogenic cell stress.

Furthermore, it remains unknown why some chemotherapeutic drugs induce ICD, some immunogenic modulation, some both and some neither. The exact mechanism of cell stress induced by a chemotherapy regimen is likely the driving factor for whether it will result in immunogenic cell stress or not, and as more is discovered about cell stress mechanisms it is possible these questions will find definitive answers.

In this review we have focused on the roles of chemotherapy in inducing immunogenic cell stress, but it should not be ignored that many other agents have been shown to have similar effects that also synergize with immuno-oncology agents, and depending on cancer indication or patient status these agents should also be considered for combination immunotherapy. In addition to chemotherapy, radiation is the best characterized inducer of immunogenic cell death (117), although recent findings have also demonstrated it is possible to induce local immunogenic cell death through photodynamic therapy (118). While high dose, stereotactic ablative body radiotherapy appears to be the most effective at inducing ICD, further research into the optimal dose, schedule and potential combinations with radiosensitizers and immuno-oncology agents is necessary (119–121). Multiple modalities of sublethal radiation including radiotherapy, external beam radiation, radiolabeled antibodies and brachytherapy have also been demonstrated to be effective inducers of immunogenic modulation (122, 123). Immunogenic modulation has also been demonstrated after treatment with endocrine deprivation agents (124, 125) and small molecule inhibitors (126–128). These findings make it clear that many cancer therapies, applied sublethally, can induce immunogenic cell stress that sensitizes tumor cells to immune killing. It should also be noted that there is potential to utilize immunogenic cell stress inducing therapies strictly as immuno-oncology agents in combination with standard-of-care therapies, for instance to abrogate the immunosuppressive effects of high-dose chemotherapy.

As cancer therapy strategies include immuno-oncology agents to greater degrees, it is becoming more crucial to identify the potential immunogenic effects of well characterized standard-of-care therapies. While much happened during the development of chemotherapy, immunotherapy is rapidly entering the age of combination therapy, which will include previously defined standard-of-care chemotherapy. By employing chemotherapy not just as an anti-cancer agent but also as immunogenic cell stress-inducing agent, clinicians will add another immuno-oncology tool to their toolbox, resulting in improved clinical success and patient recovery.

## Author Contributions

All authors contributed to the writing of this article and approved the submitted version.

## Funding

This research was supported by the Intramural Research Program of the Center for Cancer Research, National Cancer Institute (NCI), National Institutes of Health.

## Conflict of Interest

The authors declare that the research was conducted in the absence of any commercial or financial relationships that could be construed as a potential conflict of interest.

## Publisher’s Note

All claims expressed in this article are solely those of the authors and do not necessarily represent those of their affiliated organizations, or those of the publisher, the editors and the reviewers. Any product that may be evaluated in this article, or claim that may be made by its manufacturer, is not guaranteed or endorsed by the publisher.
